# A Novel Frameshifting Inhibitor Having Antiviral Activity against Zoonotic Coronaviruses

**DOI:** 10.3390/v13081639

**Published:** 2021-08-18

**Authors:** Dae-Gyun Ahn, Gun Young Yoon, Sunhee Lee, Keun Bon Ku, Chonsaeng Kim, Kyun-Do Kim, Young-Chan Kwon, Geon-Woo Kim, Bum-Tae Kim, Seong-Jun Kim

**Affiliations:** Center for Convergent Research of Emerging Virus Infection, Korea Research Institute of Chemical Technology, Daejeon 34114, Korea; emdoc96@krict.re.kr (G.Y.Y.); sunhee@krict.re.kr (S.L.); kbku@krict.re.kr (K.B.K.); chonskim@krict.re.kr (C.K.); kdkim@krict.re.kr (K.-D.K.); yckwon@krict.re.kr (Y.-C.K.); azard@krict.re.kr (G.-W.K.); btkim@krict.re.kr (B.-T.K.)

**Keywords:** frameshifting, MERS-CoV, SARS-CoV-2, coronavirus, inhibitor

## Abstract

Recent outbreaks of zoonotic coronaviruses, such as Middle East respiratory syndrome coronavirus (MERS-CoV) and severe acute respiratory syndrome coronavirus 2 (SARS-CoV-2), have caused tremendous casualties and great economic shock. Although some repurposed drugs have shown potential therapeutic efficacy in clinical trials, specific therapeutic agents targeting coronaviruses have not yet been developed. During coronavirus replication, a replicase gene cluster, including RNA-dependent RNA polymerase (RdRp), is alternatively translated via a process called -1 programmed ribosomal frameshift (−1 PRF) by an RNA pseudoknot structure encoded in viral RNAs. The coronavirus frameshifting has been identified previously as a target for antiviral therapy. In this study, the frameshifting efficiencies of MERS-CoV, SARS-CoV and SARS-CoV-2 were determined using an in vitro −1 PRF assay system. Our group has searched approximately 9689 small molecules to identify potential −1 PRF inhibitors. Herein, we found that a novel compound, 2-(5-acetylthiophen-2yl)furo[2,3-*b*]quinoline (KCB261770), inhibits the frameshifting of MERS-CoV and effectively suppresses viral propagation in MERS-CoV-infected cells. The inhibitory effects of 87 derivatives of furo[2,3-*b*]quinolines were also examined showing less prominent inhibitory effect when compared to compound KCB261770. We demonstrated that KCB261770 inhibits the frameshifting without suppressing cap-dependent translation. Furthermore, this compound was able to inhibit the frameshifting, to some extent, of SARS-CoV and SARS-CoV-2. Therefore, the novel compound 2-(5-acetylthiophen-2yl)furo[2,3-*b*]quinoline may serve as a promising drug candidate to interfere with pan-coronavirus frameshifting.

## 1. Introduction

Recent outbreaks of zoonotic coronaviruses pose a global threat to public health. Severe acute respiratory syndrome coronavirus 2 (SARS-CoV-2), an etiological agent of the newly emerged coronavirus disease-19 (COVID-19), has spread worldwide, causing enormous human casualties [1]. In addition to SARS-CoV-2, cases of Middle East respiratory syndrome coronavirus (MERS-CoV), which was first identified in Saudi Arabia in June 2012 [2], are still being reported in the Middle East region, including Saudi Arabia, the United Arab Emirates, and Qatar, according to a recent case report in March 2021 (WHO, Geneva, Switzerland, 2021) [3].

These zoonotic viruses (i.e., SARS-CoV, SARS-CoV-2 and MERS-CoV) belong to the genus *Betacoronavirus*, family *Coronaviridae*, order *Nidovirales* [4]. CoVs are enveloped and have the largest genome among RNA viruses. The coronavirus (CoV) genome consists of a single-stranded, positive-sense RNA approximately 26–32 kb in length [5]. The genome has a 5′ untranslated region (UTR), open-reading frames (ORFs), and a 3′ UTR. The 5′-proximal two-thirds of the entire genome contains ORFs 1a and 1b, which are translated into two large replicase polyproteins. The other one-third of the genome encodes structural and accessory proteins [6].

ORF1a encodes polyprotein 1a (pp1a), while ORF1a and ORF1b together encode poly protein 1ab (pp1ab) through programmed −1 ribosomal frameshift (−1 PRF). The −1 PRF is an alternative mechanism for translation whereby the ribosome stalls on the mRNA secondary structure, shifts the ribosome reading frame back to one nucleotide, and continues the translation. As ORF1b lacks its own independent translation initiation site, ORF1b translation requires −1 PRF at the junction of the ORF1a/ORF1b overlap region [7,8]. After translation, the two large polyproteins, pp1a and pp1ab, are cleaved by viral protease-mediated proteolysis and produce nonstructural proteins (nsps) 1–11 encoded in ORF1a and nsp12–16 in ORF1b. The nsps comprise a replication-transcription complex (RTC) and carry out viral RNA replication, transcription, and translation. Among them, nsp12–16 plays an important role in RTC, that is RNA-dependent RNA polymerase (RdRp or nsp12), helicase (HEL or nsp13), exonuclease (ExoN or nsp14), endonuclease (NendoU or nsp15), and methyltransferase (2´-O-MT or nsp16) [9,10,11,12].

In reverse genetics studies on CoVs, it has been reported that ORF1b-encoded proteins are essential for viral RNA replication. Work on the infectious bronchitis virus (IBV), a highly infectious avian CoV, demonstrated that point mutations in nsp13 in Vero cell-adapted IBV clones cause lethal viral infection in Vero cells [13]. A study using infectious clones of SARS-CoV mutants with in-frame deletions at nsp14, nsp15, and nsp16 suggested that ExoN, NendoU, and 2´-O-MT are essential for efficient viral RNA replication [14]. Hence, the translation mechanism via −1 PRF is essential for the CoV life cycle because of the crucial role of the ORF1b replicase protein in viral RNA replication.

Other viruses utilize −1 PRF to control the gene expression involved in replication and viral pathogenesis. During human immunodeficiency virus 1 (HIV-1) infection, the pol gene, which is downstream of the gag gene, is translated by frameshifting and the gag to gag-pol ratio is strictly regulated for efficient viral replication, assembly, and infectivity [15,16,17]. In West Nile virus (WNV) and Japanese encephalitis virus (JEV) serogroups, NS1′ (nonstructural) proteins associated with viral neuro-invasiveness are produced by frameshifting [18,19]. In alphaviruses such as the Sindbis virus (SINV), Semliki Forest virus (SFV), and Chikungunya virus (CHIKV), the generation of TransFrame (TF) protein by −1 PRF has recently been discovered [20]. TF proteins are known to be important for alphavirus assembly and neuropathogenesis [21,22]. Since replicase proteins (nsp 12–16) comprising RdRp and helicase are translated by −1 PRF in coronaviruses, the frameshifting efficiency affects genomic RNA replication and sub-genomic RNA production [23]. Furthermore, the ratio between ORF1a and ORF1b proteins affects viral propagation and infectivity [24].

In general, frameshifting signals of CoVs are composed of two elements: conserved heptameric slippery sequences (UUUAAAC), followed by RNA secondary structure (a pseudoknot RNA structure) [7,8]. These components contribute to an efficient frameshifting process, and disrupting the pseudoknot structure leads to the malfunctioning of this process. In mutagenesis studies on the frameshifting of SARS-CoV, deletions and mutations in the pseudoknot structure have negative effects, inhibiting or abolishing frameshifting [23,25]. The RNA pseudoknot structure of the −1 PRF signal is highly conserved among coronaviruses, rendering −1 PRF an attractive antiviral target [25]. It has been shown that antisense peptide nucleic acids (PNAs) targeting the −1 PRF signal inhibits efficient SARS-CoV replication, disrupting the pseudoknot structure [26].

In this study, we determined the frameshifting efficiencies of SARS-CoV, SARS-CoV-2, and MERS-CoV, and searched for small molecules that inhibit the −1 PRF of MERS-CoV. We discovered a novel compound that efficiently suppressed viral propagation via inhibition of −1 PRF, suggesting that the −1 PRF of coronaviruses is a potential target for antiviral therapy.

## 2. Materials and Methods

### 2.1. Cell Culture and Virus Infection

Huh7 and A549 cells were purchased from American Type Culture Collection (ATCC, Manassas, VA, USA). Huh7, A549 and HEK293 cells were maintained in Dulbecco’s modified Eagle medium with high glucose (DMEM) with 10% fetal bovine serum (FBS, Gibco) and 1% penicillin/streptomycin (Invitrogen, Waltham, MA, USA). The Middle East respiratory syndrome coronavirus (MERS-CoV) strain isolated from a Korean patient (GenBank accession number KT029139.1) was kindly provided by the Korea Centers for Disease Control and Prevention [27]. All experiments with infectious viruses were conducted in a biosafety level 3 (BSL-3) laboratory at the Korea Research Institute of Chemical Technology (KRICT, Daejeon, Korea).

### 2.2. Plasmids

Reporter plasmids containing the frameshifting signals were generated using the dual-luciferase vector system (pSGDluc), which was developed by Loughran et al. to avoid assay distortions from fused dual luciferase reporters [28]. The −1 PRF sequences of MERS-CoV (accession number KT029139.1), SARS-CoV-1 (accession number AY278741), SARS-CoV-2 (accession number NC_045512.2), HCoV-229E (accession number NC_002645), HCoV-HKU1 (accession number NC_006577), HCoV-NL63 (accession number NC_005831), and HCoV-OC43 (accession number NC_006213.1) were obtained from GenBank database. The −1 PRF sequences of MERS-CoV, SARS-CoV-1, and SARS-CoV-2 were synthesized and inserted between *Renilla* luciferase and firefly luciferase genes in the pSGDluc vector. Further, −1 PRF sequences were inserted in a manner that firefly luciferase is only expressed with *Renilla* luciferase when −1 PRF occurs. Then, the −1 PRFs with reporter genes were subcloned into a lentiviral vector, specifically the pLenti CMV/TO Puro empty (w175-1) vector. Lentiviruses were generated using packaging plasmids (pMD2.G and psPAX2). A549 cells were transduced with lentiviruses, and the cells stably expressing reporter genes were selected using puromycin. Positive and negative controls that did not contain −1 PRF signals were also generated. In control constructs, *Renilla* and firefly luciferase genes were linked in-frame for a positive control or out of frame for a negative control. Thus, the positive control expresses both *Renilla* and firefly luciferases, whereas the negative control only expresses *Renilla* luciferase.

### 2.3. Chemical Library Screening and Dual-Luciferase Assays

Small molecule libraries (each dissolved in 100% DMSO) were kindly provided by the Korea Chemical Bank [29] at the Korea Chemical Institute of Chemical Technology (KRICT, Daejeon, Korea). A549 cells stably expressing reporter genes were seeded on a 384-well plate using a Multidrop™ 384 reagent dispenser (Thermo Fisher Scientific, Waltham, MA, USA). The cells were treated with 5 µM of each chemical compound for 24 h. The luciferase activities were measured using a Dual-Glo^®^ luciferase assay system (Promega, E2940, Madison, WI, USA) and a Synergy H1 microplate reader (BioTek, Winooski, VT, USA). Frameshift efficiency was determined by measuring the ratio of *Renilla* luciferase activity to firefly luciferase activity. The positive control was set to 100%, and the negative control was set to 0%. Relative frameshifting ratio was calculated by comparing relative frameshifting ratio (*Renilla* luciferase activity to firefly luciferase activity) of the indicated samples to the ratio of the DMSO-treated or untreated samples, which was set to 100%. IC_50_ values were determined by a sigmoidal dose-response (variable slope) curve using GraphPad Prism 8 (GraphPad Software, LLC, San Diego, CA, USA) software.

### 2.4. Cell Viability

The viability of cells grown on a 96-well plate was measured using a WST-1 reagent kit (D-Plus™ CCK Cell Viability Assay Kit, Dongin Biotech, Seoul, Korea) and a Synergy H1 microplate reader (BioTek). Cells were treated with increasing concentrations of the chemical compounds for 2 days.

### 2.5. Quantitative RT-PCR

For viral infections, Huh7 cells grown on a 96-well plate were incubated with 0.02 MOI of MERS-CoV for 1 h. The cells were washed twice with serum-free media and then grown in fresh media with or without chemical compounds for 2 days. Viral RNA was extracted from cell culture supernatants using the QIAamp Viral RNA Mini Kit (Qiagen, Germany) according to the manufacturer’s protocol. To detect the MERS-CoV genome, primers targeting the MERS-CoV nucleocapsid (N) gene (28,748–28,814 nucleotides of MERS-CoV) were used: forward primer 5′- GGG TGT ACC TCT TAA TGC CAA TTC -3′ and reverse primer 5′- TCT GTC CTG TCT CCG CCA AT -3′. RNA transcribed from the MERS-CoV nucleocapsid gene using the MEGAscript™ T7 transcription kit was used as a standard for qRT-PCR. qRT-PCR was performed with the One Step PrimeScript III RT-qPCR mix (Takara) reagent using the QuantStudio 3 real-time PCR system (Applied Biosystems).

### 2.6. Statistical Analysis

Statistical analyses were performed using GraphPad Prism 8 software (GraphPad Software, LLC). *p* values of less than 0.05 were considered statistically significant. Data produced by qRT-PCR, luciferase assays, and cell viability assays are presented as mean values, with error bars showing the standard deviations from three independent experiments, unless otherwise specified.

## 3. Results

### 3.1. Novel Compound Inhibiting MERS-CoV Frameshifting

**Figure 1:**
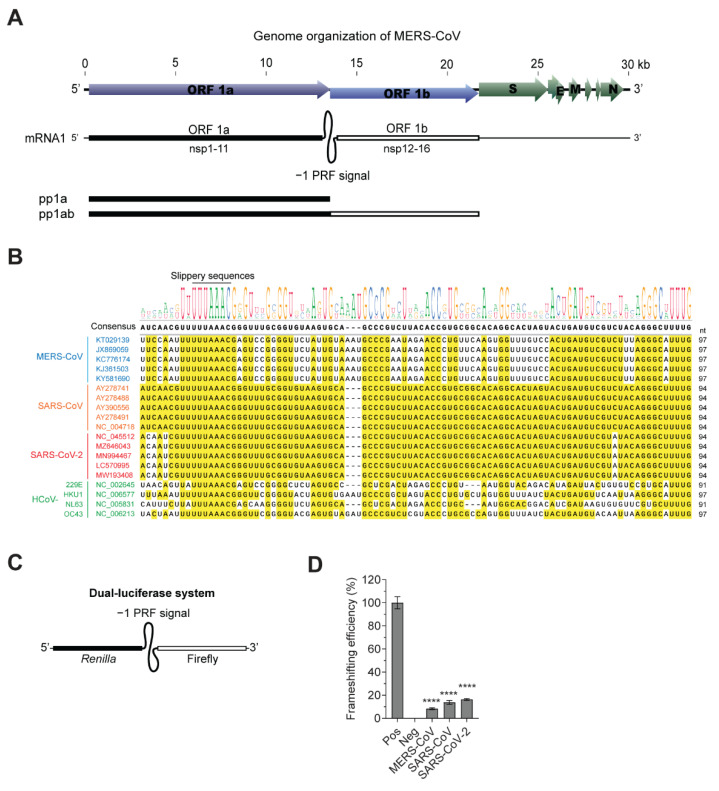
Frameshifting ratio of MERS-CoV, SARS-CoV-1, and SARS-CoV-2. (**A**) Schematic diagram of the genomic organization of MERS-CoV. (**B**) Alignment of the full sequences of −1 PRF signals of MERS-CoV, SARS-CoV, SARS-CoV-2, HCoV-229E, HCoV-HKU1, HCoV-NL63, and HCoV-OC43. Slippery sequences (UUUAAAC) are indicated above the aligned sequences. (**C**) Schematic diagram of a reporter system. (**D**) Frameshifting efficiencies of MERS-CoV, SARS-CoV, and SARS-CoV-2. Positive control expressing *Renilla* and firefly luciferases fused without −1 PRF signal, was set to 100% and negative control only expressing *Renilla* luciferase to 0%. Pos, positive control; Neg, negative control. **** *p* < 0.0001 vs. positive control (one-way analysis of variance (ANOVA) with Dunnett’s multiple comparisons test).

MERS-CoV ORF 1b contains a replicase gene cluster, which consists of key components required for viral replication, including RNA-dependent RNA polymerase (RdRp) and helicase. ORF1b is translated by −1 PRF, followed by ORF1a expressing polyprotein 1ab (pp1ab) (Figure 1A). Presently, seven coronaviruses were identified to infect humans: MERS-CoV, SARS-CoV, SARS-CoV-2, HCoV-229E, HCoV-HKU1, HCoV-NL63, and HCoV-OC43. The −1 PRF signals of these human-infecting coronaviruses have approximately 91–97 nucleotides and contain slippery sequences (UUUAAAC), where frameshifting occurs. Nucleotide sequences of the −1 PRF signal of human-infecting coronaviruses isolated at different locations were aligned to compare the sequences. While the −1 PRF sequences of SARS-CoV and SARS-CoV-2 are highly homologous, those of SARS-CoV and MERS-CoV are less conserved (Figure 1B).

To determine frameshifting efficiency, we first established lentivirus-based dual-luciferase reporter system containing −1 PRF signals. The full sequences of −1 PRF signals corresponding to the pseudoknot structure depicted in Figure 1 and Table 1 were inserted between *Renilla* and firefly luciferase genes such that firefly luciferase genes were translated only when frameshifting occurred through the pseudoknot secondary RNA structures in mRNAs decoded in −1 PRF signals (Figure 1C). The frameshifting efficiencies were measured in A549 lung epithelial cells stably expressing reporter genes containing −1 PRF signals. The frameshifting efficiencies of MERS-CoV, SARS-CoV and SARS-CoV-2 were 8.4%, 13.8% and 16.3%, respectively (Figure 1D). The frameshifting efficiency of SARS-CoV was consistent with the previously determined frameshifting efficiency (14–15%) in mammalian translation system [23,25]. The relatively lower frameshifting efficiency of MERS-CoV compared to SARS-CoV and SARS-CoV-2 may be associated with sequence variation and pseudoknot structure.

**Figure 2:**
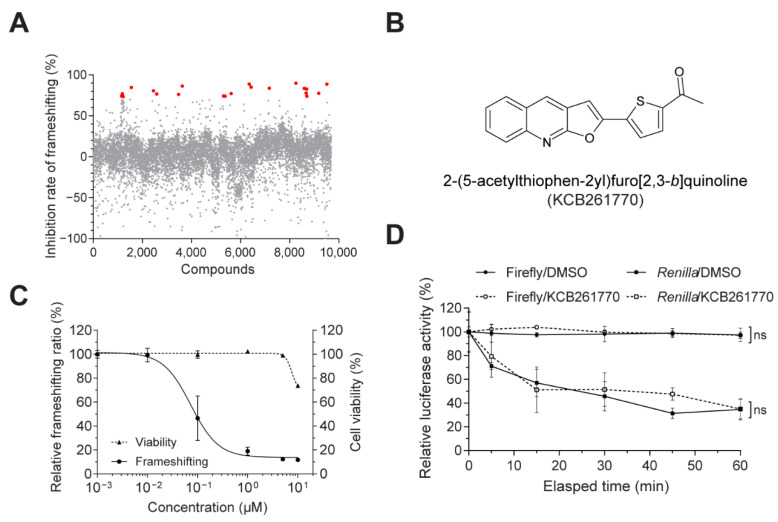
A novel compound efficiently inhibiting MERS-CoV frameshifting. (**A**) Screening of compounds inhibiting the MERS-CoV −1 PRF frameshifting. Chemical compounds with inhibitory effects over 70% are indicated with red dots. (**B**) Chemical structure of the selected compound, 2-(5-acetylthiophen-2yl)furo[2,3-*b*]quinoline (KCB261770). (**C**) The relative frameshifting ratio of MERS-CoV −1 PRF (solid line, left *y*-axis) and cell viability (dashed line, right *y*-axis) were tested with increasing concentrations of KCB261770. (**D**) Cell lysates harboring MERS-CoV −1 PRF plasmids were incubated with compound KCB261770 for 60 min. The firefly and *Renilla* luciferase activities relative to the initial time points were monitored. The luciferase activities of the samples, including the compound-treated samples, were compared to those of the luciferase activities of the DMSO-treated samples at 0 time, which was set to 100%. DMSO was used as a negative control. ns; not significant (two-way ANOVA with Sidak’s multiple comparisons test).

Using the established evaluation system for −1 PRF efficiency, we screened chemical compounds that inhibit MERS-CoV frameshifting in the chemical libraries of the Korea Chemical Bank. Among the chemical libraries (total: 640,000 chemicals), we initially chose a representative library (6566 chemical compounds), a natural compound library (1019 chemical compounds), and a clinically applied library (2104 chemical compounds). Compounds that exhibited over 70% inhibition at a concentration 5 μM of were selected (Figure 2A). Among these compounds, compounds with significantly reduced *Renilla* luciferase activity (i.e., general translation) were removed. Six compounds were selected, and their IC_50_ values and cell viabilities (WST assays) were determined with various concentrations of compounds (0.01–10 μM). Of that group, compound KCB261770 showed the most promising inhibitory effects (IC_50_ = 0.07 μM, CC_50_ = 18.5 μM) and was chosen for further study (Figure 2B,C). Compound KCB261770, 2-(5-acetylthiophen-2yl)furo [2,3-*b*]quinoline, is a newly synthesized furo[2,3-*b*]quinoline with a molecular weight of 294.3 [30]. Although furo-quinoline occurs naturally [31,32], diverse furo-quinoline derivatives have also been synthesized [30,33] owing to their pharmacological and biological properties including anti-inflammatory [34,35], anti-malarial [36], and antimicrobial activities [37]. The inhibitory effects, cell viabilities, IC_50_ values, and chemical structures of the rest are shown in Figure S1.

To exclude the possibility of a false positive, such as direct luciferase inhibition or luminescence-interfering activities, direct inhibition of luciferase activity by compound KCB261770 was monitored in cell lysates harboring MERS-CoV −1 PRF plasmids. The cell lysates were incubated with or without 5 μM KCB261770. Accordingly, firefly and *Renilla* luciferase activities were measured for 60 min. Both firefly and *Renilla* luciferase activities were not significantly affected by compound KCB261770 (Figure 2D). Therefore, it is clear that compound KCB261770 does not directly inhibit luciferase activity.

**Figure 3:**
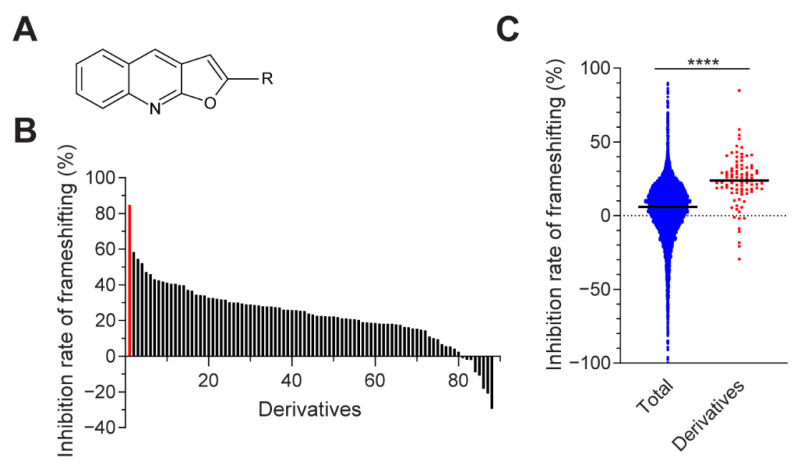
Inhibitory effects of furo[2,3-*b*]quinoline derivatives on MERS-CoV frameshifting. (**A**) Chemical structure of furo[2,3-*b*]quinoline derivatives. (**B**) The inhibition rate of frameshifting among a total of 88 derivatives, including compound KCB261770 indicated in red, were determined. (**C**) Inhibition rate of frameshifting was compared between the initial library (blue dots) and furo[2,3-*b*]quinoline derivatives (red rectangles). The thick bars in the center represent the median values. **** *p* < 0.0001 (unpaired *t*-test with Welch’s correction).

We further tested another 87 derivatives of furo[2,3-*b*]quinolines from the Korea Chemical Bank Library (Figure 3A,B). Among the tested derivatives, compound KCB261770 featured the most prominent inhibitory effect compared to the other derivatives (Figure 3B). We compared the overall inhibitory effects of the initial library (9689 chemicals) and derivatives (88 chemicals). The median value of the inhibition rate of the furo[2,3-*b*]quinoline derivatives showed 19.83% greater inhibitory effects than that of the initial library, suggesting that the furo[2,3-*b*]quinoline backbone had basal inhibitory effects (Figure 3C). In the meantime, compound KCB261770, which has an acetyl-thiophene group of 2-(5-acetylthiophen-2yl)furo[2,3-*b*]quinoline, showed drastically enhanced inhibitory effects compared to the other derivatives. Therefore, the acetyl-thiophene group may serve as an important determinant factor for inhibitory effects on frameshifting. We also compared the chemical structure of KCB261770 with previously reported compounds that have inhibitory effects on frameshifting, including triazole [38], anisomycin [39], MTDB [40], and a novel compound named 43 [41]. KCB261770 did not have backbone structures similar to those of other small molecules that have been reported (Table S1).

### 3.2. Inhibition of the Frameshifting of Zoonotic Coronaviruses by Compound KCB261770

In order to exclude indirect effects, such as inhibition of general transcription or translation, we compared how the two luciferase activities linked by the frameshifting signal in our assay system were impacted by compound KCB261770, 6-thioguanine (a DNA synthesis inhibitor), and cycloheximide (CHX; a general inhibitor of translation elongation). 6-thioguanine, a well-studied purine analog, inhibits de novo purine synthesis and is incorporated into DNA and RNA, leading to disruption of DNA synthesis and cytotoxicity [42,43]. CHX binds to the E site of the 60S ribosome subunit of the translation machinery and inhibits translation by blocking the translocation step during elongation [44].

**Figure 4:**
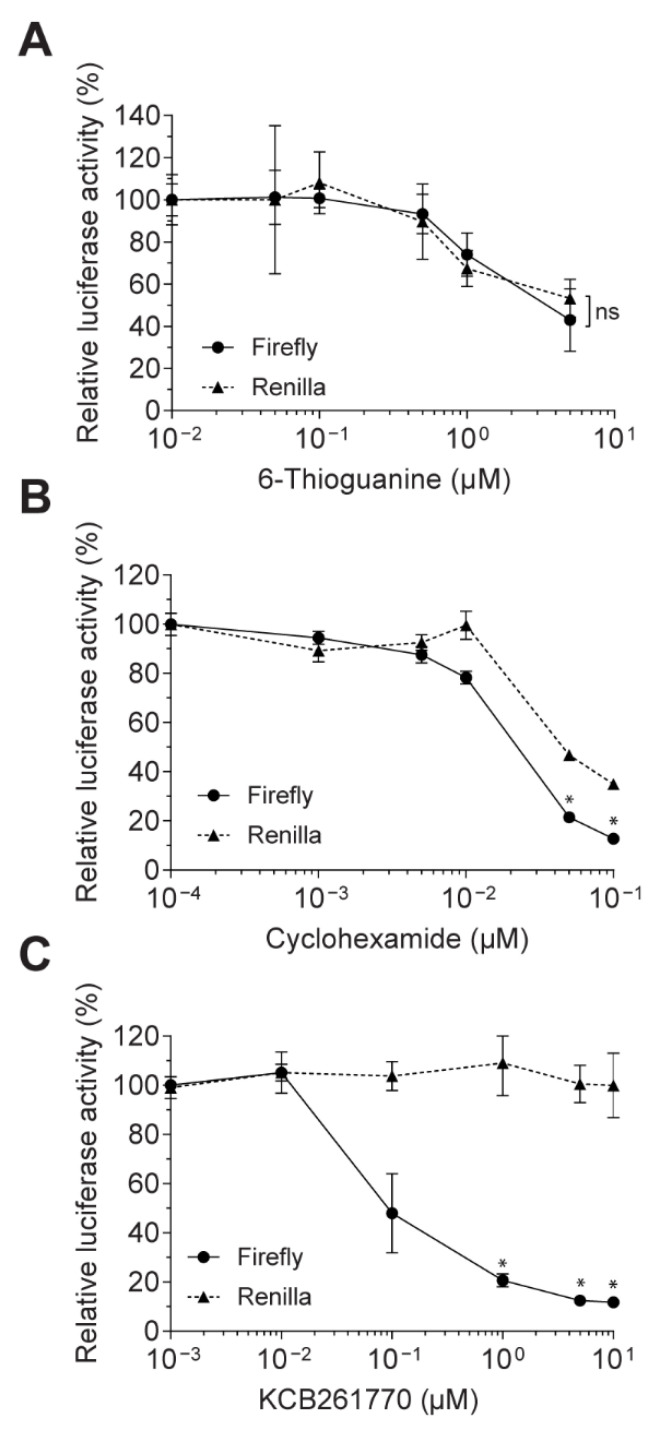
Frameshifting inhibition by compound KCB261770 without decreasing cap-dependent translation. (**A**–**C**) Relative firefly and *Renilla* luciferase activities were measured in the cells harboring MERS-CoV −1 PRF plasmids treated with increasing concentrations of 6-thioguanine (**A**), cycloheximide (CHX; **B**), and compound KCB261770 (**C**). Circles with solid lines indicate firefly luciferase activities and triangles with dashed lines *Renilla* luciferase activities. * *p* < 0.05, ns; not significant (two-way ANOVA with Sidak’s multiple comparisons test).

Firefly and *Renilla* luciferase activities were measured in cells harboring reporter plasmids containing the MERS-CoV −1 PRF. 6-thioguanine inhibited both firefly and *Renilla* luciferase activities in a dose-dependent manner without decreasing the frameshifting ratio, suggesting that it was not inhibited by the cytotoxicity induced by 6-thioguanine (Figure 4A). CHX also inhibited both firefly and *Renilla* luciferase activities in a dose-dependent manner. Unlike 6-thioguanine, firefly luciferase activity was inhibited more sensitively than *Renilla* luciferase activity, resulting in 63.5% inhibition of the frameshifting ratio at a 0.1 μM concentration (Figure 4B). It is likely that CHX blocks the translocation step, where frameshifting is involved. In contrast, when treated with compound KCB261770, only firefly luciferase activity was inhibited in a dose-dependent manner without a decrease in *Renilla* luciferase activity, resulting in a drastic reduction in the frameshifting ratio (Figure 4C). These data clearly show that compound KCB261770 inhibits the frameshifting of MERS-CoV −1 PRF, but not by indirect inhibition of translation elongation or other cytotoxicity.

**Figure 5:**
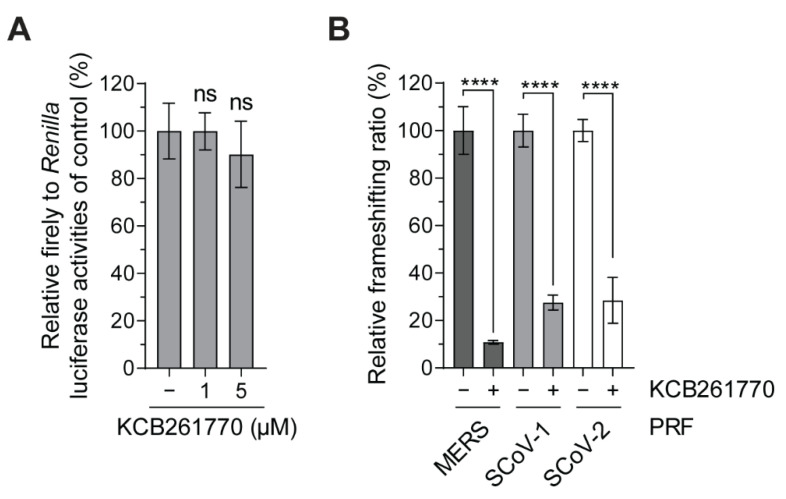
Comparison of the inhibitory effects of compound KCB261770 on −1 PRFs of SARS-CoV, SARS-CoV-2, and MERS-CoV. (**A**,**B**) Frameshifting ratio relative to the untreated control was measured with or without compound KCB261770 (5 μM) in the indicated −1 PRFs. Frameshifting ratio was measured in the control cells, which does not have a PRF signal (**A**) or in the cells harboring coronavirus −1 PRFs (**B**). Frameshifting ratio of the each indicated PRF without treatment was set to 100%. MERS; MERS-CoV PRF, SARS-1; SARS-CoV PRF, SARS-2; SARS-CoV-2 PRF. **** *p* < 0.001, ns; not significant vs. control (one-way ANOVA with Tukey’s multiple comparisons test).

As described in Figure 1, while the −1 PRF signals of SARS-CoV and SARS-CoV-2 share almost the same sequences [45], MERS-CoV has less conserved sequences [46]. Thus, we further investigated whether compound KCB261770 is also effective for the other frameshifting signals. The inhibitory effects of compound KCB261770 on MERS-CoV, SARS-CoV, and SARS-CoV-2 frameshifting at a 5 μM concentration were examined. Consistent with previously reported inhibitory effects of compound KCB261770 (Figure 2C), it dramatically inhibited MERS-CoV frameshifting by 91.6% at a concentration of 5 μM (Figure 5B). In the meantime, inhibition of the frameshifting of SARS-CoV and SARS-CoV-2 by compound KCB261770 was 72.4% and 71.6%, respectively (Figure 5B). The SARS-CoV-2 frameshifting was reduced by KCB261770 treatment in a dose-dependent manner (1 μM and 5 μM) and its IC_50_ was approximately 0.54 μM (Figure S2A). The control cells that did not have the frameshifting signal were not significantly affected by KCB261770 (Figure 5A). It also did not significantly affect proliferation of parental A549 cells and their morphologies (Figure S2B,C). Therefore, KCB261770 exhibited more selective inhibition of the MERS-CoV −1 PRF signal than the SARS-CoV and SARS-CoV-2 −1 PRF signals.

To compare the selectivity and effectiveness of the compound with other small molecules, we further tested whether the previously known compounds that inhibit -1 ribosomal frameshifting efficiencies can interfere with MERS-CoV −1 PRF. Anisomycin, a peptidyl-transferase inhibitor, is known to inhibit the frameshifting of M_1_ dsRNA virus [39] and HIV [47] (Table S1). Cell viability and frameshifting ratios were determined. The inhibitory effect on the frameshifting was measured up to a concentration of 50 nM, which did not show significant cellular toxicity. The frameshifting efficiency was not significantly reduced by anisomycin treatment (Figure S3), supporting the selective inhibition of MERS-CoV frameshift by KCB261770.

### 3.3. Compound KCB261770 Efficiently Suppresses MERS-CoV Propagation

**Figure 6:**
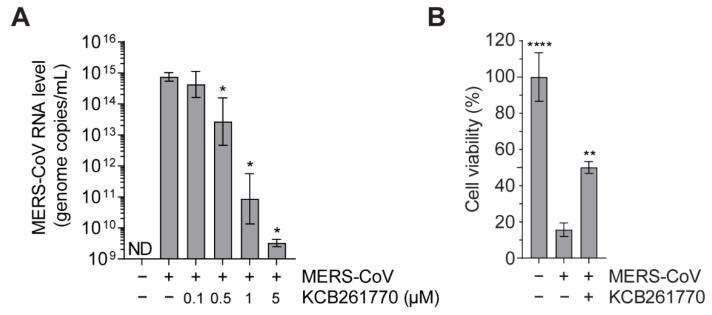
Suppression of MERS-CoV propagation by compound KCB261770. (**A**) Huh7 cells infected with 0.02 MOI of MERS-CoV were treated with increasing concentrations of compound KCB261770. At 2 dpi, genome copy number of MERS-CoV RNA in the media were determined by qRT-PCR using primers targeting MERS-CoV N gene. ND; not detected. (**B**) Cell viability was measured at 2 dpi with or without treatment of compound KCB261770 (5 μM). * *p* < 0.05, ** *p* < 0.01, **** *p* < 0.0001 vs. control (MERS-CoV-infected cells without treatment) (one-way ANOVA with Dunnett’s multiple comparisons test).

Recently, some chemical compounds targeting the frameshifting of HIV-1 and CoVs have been developed to suppress viral propagation [38,40,41,45,48]. However, only few of these have been confirmed with live viruses. Here, we examined whether KCB261770 could suppress viral dissemination of MERS-CoV. Huh7 cells infected with MERS-CoV were treated with increasing concentrations of compound KCB261770. In untreated MERS-CoV-infected cells, high levels of MERS-CoV RNA (approximately 1.1 × 10^15^ genome copies/mL) in the media were detected. In contrast, compound KCB261770 treatment dramatically decreased viral RNA levels in the media in a dose-dependent manner (approximately 3.7 × 10^9^ genome copies/mL at 5 μM), suggesting that the production of progeny viruses was suppressed by the treatment (Figure 6A). While extensive cell death was observed in untreated MERS-CoV-infected cell, virus-induced cell death was also reduced significantly after treatment with 5 μM KCB261770 compared to that in untreated cells (Figure 6B). The underlying mechanism of action of the compound was to interfere with the expression level of replicase gene cluster instead of suppressing the activity of viral proteins, which have immediate antiviral effects. Thus, it seems that inhibiting frameshifting might have fewer protective effects against virus-induced cell death by the initially infected viruses, resulting in the limited recovery of virus-infected cells at an early stage of infection despite the dramatic decrease in progeny virus production.

Overall, our data clearly demonstrate that compound KCB261770 suppresses MERS-CoV propagation via inhibition of frameshifting, corroborating that the frameshifting of coronaviruses can serve as a potential target for the development of therapeutic agents.

## 4. Discussion

Studies on the effective regulation of biological functions of RNAs by small molecules have been recently accumulating [49,50]. In particular, viral RNAs, which have different features from those of human beings, have been of great interest as potential therapeutic targets. The 5′- and 3′- untranslated regions (UTRs), internal ribosome entry sites (IRESs), and PRF signals in viral RNAs are good examples. Viral UTRs provide binding sites for replicase proteins to copy their genomes [51]. IRES elements allow initiation of cap-independent translation of viral proteins [52]. PRFs enable the alternative translation of viral proteins [53]. Our research has focused on the regulation of the coronavirus frameshifting by small molecules to control viral propagation.

In this study, the frameshifting signals of SARS-CoV, SARS-CoV-2, and MERS-CoV were compared within the same reporter system stably expressed in human lung epithelial cells. The frameshifting efficiencies of SARS-CoV and SARS-CoV-2 were similar to those of highly conserved nucleotide sequences. This is consistent with recent reports showing functional conservation of the −1 PRF of SARS-CoV-2 analyzed by NMR spectroscopy or small-angle X-ray scattering profiles [45,54]. The PRF efficiency of SARS-CoV was previously reported by Plant et al. and Baranov et al. to have a 10–16% rate in rabbit reticulocyte lysates and in human cells [23,24,25,55]. The frameshifting ratio of SARS-CoVs determined in our system was consistent with that of their group. However, the frameshifting ratios of SARS-CoV-2 and MERS-CoV were slightly lower than those of other groups [45,56]. Kelly et al. reported a 20–30% of frameshifting rate of the SARS-CoV-2, and Hu et al. reported 14% of MERS-CoV. The PRF efficiency may differ depending on the reporter system or translation system in various cell lines [25]. Kelly et al. and Hu et al. measured PRF efficiencies in a cell-free assay system using rabbit reticulocyte lysate with in vitro-transcribed RNAs or in human cell culture systems with transient transfection (HEK or HeLa) [45,56]. We established a stable human lung cell line harboring the reporter system, which would more likely mimic natural translation in human lung cells. When the frameshifting ratios of SARS-CoV, SARS-CoV-2, and MERS-CoV were compared simultaneously in our system, the SARS-CoVs frameshifting efficiency was slightly higher than that of MERS-CoV, with some nucleotide variations.

The frameshifting efficiency confers genomic RNA replication and sub-genomic RNA production, which, in turn, modulates viral propagation and infectivity (Plant et al., 2010, Plant et al., 2013). We previously showed that the inhibition of the frameshift correlates well with the decrease in SARS-CoV replication using antisense peptide nucleic acids (PNAs) specifically targeting the −1 PRF signal (Ahn et al., 2011). In this study, we found a novel small molecule that strongly inhibited the frameshifting of MERS-CoV, which was confirmed in the virus-infected cells. The compound was also effective in the frameshifting of SARS-CoV or SARS-CoV-2, due to the structural similarities between the PRF signals of MERS-CoV and SARS-CoV [46]. Thus, it might have antiviral effects against a broad range of CoVs owing to the conserved pseudoknot structures with more selectivity towards MERS-CoV frameshifting.

For further development of antiviral agents targeting frameshifting, it is necessary to identify the underlying mechanisms by which the novel compound interferes with frameshifting. The frameshifting event occurs via interaction between the ribosomal complex and the pseudoknot structure of the −1 PRF signal [53]. A recent study on the RNA structure of SARS-CoV-2 also revealed the interaction of the pseudoknot structure with the ribosomal complex and 18S rRNA [57]. In the case of EMCV, the frameshifting is mediated by viral protein 2A, which binds to PRF signals [58]. However, such viral proteins mediating frameshifting have not yet been reported in coronaviruses. As general translation was not affected by KCB261770, it is highly likely that KCB261770 might interfere with the interaction between RNA structure and translation complex by binding to the non-active sites of ribosomal complexes where the interaction occurs, but not the functionally active sites of ribosomal complexes such as A- or P-sites of ribosomes and peptidyl transferase. It is also possible that KCB261770 might modulate the frameshifting ratio by directly binding to the RNA element, modifying the secondary structure of the RNA, which in turn makes it unable to interact with the ribosomal complex. Although further studies are needed to dissect the exact molecular mechanisms, our studies show that the frameshifting signals are viable targets for the antiviral therapeutics, providing a novel compound as a promising starting point to develop a potential small molecule drug candidate that interferes with coronavirus frameshifting. This approach has some advantages over traditional antivirals that target viral proteins. It can exhibit multiple inhibitory effects by modulating the expression levels of several viral proteins under the PRF signal (e.g., replicase gene cluster in coronaviruses). In addition, highly conserved PRF sequences and their secondary RNA structures make this approach less vulnerable to viral mutations [25]. Otherwise, mutant viruses would readily be resistant to the drugs. Therefore, it provides more benefits for combinatorial therapies with two different mechanisms (e.g., targeting RdRp and PRF), thereby providing an improved therapeutic approach to treat pan-coronaviruses including viral mutations and variants.

Although some furo-quinoline-containing plant extracts have been used in traditional medicine [31,37], a limited number of studies has been conducted in the context of their safety and the underlying metabolic processes they induce. A recent study by Huang et al. examined the in vivo metabolite profiling of skimmianine, a furo-quinoline mainly present in a flowering plants of the Rutaceae family [59]. When skimmianine was orally administered in rats, it was extensively metabolized and excreted through urine in the form of metabolites. Thus, the metabolic profiling of the novel compound and antiviral activities of its metabolites should also be carefully analyzed for therapeutic development. Further studies are warranted to the development of more specific antiviral therapeutics targeting the frameshifting of CoVs.

## Figures and Tables

**Table 1:** Sequences of frameshift signals used in this study.

Viruses	Inserted PRF Sequences (5′–3′) ^a^	Accession Number and Position
MERS-CoV	TTCCAATTTTTTAAACGAGTCCGGGGTTCTATTGTAAATGCCCGAATAGAACCCTGTTCAAGTGGTTTGTCCACTGATGTCGTCTTTAGGGCATTTG	KT029139.1 13418–13514 nt (97 nucleotides)
SARS-CoV	ATCAACGTTTTTAAACGGGTTTGCGGTGTAAGTGCAGCCCGTCTTACACCGTGCGGCACAGGCACTAGTACTGATGTCGTCTACAGGGCTTTTG	AY278741 13,382–13,477 nt (94 nucleotides)
SARS-CoV-2	ACAATCGTTTTTAAACGGGTTTGCGGTGTAAGTGCAGCCCGTCTTACACCGTGCGGCACAGGCACTAGTACTGATGTCGTATACAGGGCTTTTG	NC_045512.2 13,452–13,547 nt (94 nucleotides)

^a^ Slippery sequences are underlined.

## Supplementary Materials

The following are available online at https://www.mdpi.com/article/10.3390/v13081639/s1, Figure S1. Potential chemical compounds inhibiting MERS-CoV frameshifting, Figure S2. Dose-dependent inhibition of the SARS-CoV-2 frameshifting by KCB261770, Figure. S3. The effect of anisomycin on MERS-CoV frameshifting, Table S1. Small molecules that have been reported previously.
