# Supplementary material for: A Novel Frameshifting Inhibitor Having Antiviral Activity against Zoonotic Coronaviruses
Source: Viruses. 2021 Aug 18;13(8):1639. doi: 10.3390/v13081639 (PMC8402677; doi:10.3390/v13081639)
Supplement: Supplementary file 1 [file viruses-13-01639-s001.zip › viruses-1316449-supplementary.pdf]

Supplemental materials

Supplemental figure legends

Fig. S1. Potential chemical compounds inhibiting MERS-CoV frameshifting.

(A-E) The relative frameshifting ratio of MERS-CoV -1 PRF (solid line, left y-axis), cell viability (dashed line, right y-axis), and IC_{50} value were shown with increasing concentrations of each compound. Chemical structure of each compound was shown on the right panel. Compound A: 4-[2-Amino-3-cyano-5-oxo-4-(2-thienyl)-5,6,7,8-tetrahydro-1(4H)-quinolinyl]-3-thia-1-azatricyclo[5.2.2.02,6]undeca-2(6),4-diene-5-carbonitrile; compound B: tert-butyl [4-(1-methyl-2-oxo-1,2-dihydroquinolin-3-yl)phenoxy]acetate; compound C: 2-[(1r,4r)-4-[4-(6-{[(tert-butoxy)carbonyl]amino}-7-oxo-5H,6H,7H-pyrrolo[3,4-b]pyridin-2-yl)phenyl]cyclohexyl]acetic acid; compound D: 3-{[5-(1-Naphthyl)-1,3,4-oxadiazol-2-yl]sulfanyl}propanoic acid; compound E: 4-(4-Methoxy-2,3,6-trimethylbenzyl)-1,8-dioxa-4,11-diazaspiro[5.6]dodecane

Fig. S2. Dose-dependent inhibition of the SARS-CoV-2 frameshifting by KCB261770.

(A) Frameshifting ratio relative to the untreated control was measured with increasing concentration of compound KCB261770 in the SARS-CoV-2 -1 PRF. (B) Cell proliferation of parental A549 cells was measured using CellTiter 96® AQueous one solution cell proliferation assay (Promega, USA). Increasing concentrations of the chemical compounds were used to treat the cells for 2 days. (C) Images of cell morphologies were taken by Nikon Elipse T*i* microscope system. Frameshifting ratio or cell proliferation without treatment was set to 100%. **$p<0.01$, ***$p<0.001$ vs. control (one-way ANOVA with Dunnett's multiple comparisons test).

Fig. S3. The effect of anisomycin on MERS-CoV frameshifting.

The relative frameshifting ratio of MERS-CoV -1 PRF (solid line, left y-axis) and cell viability (dashed line, right y-axis) were measured with increasing concentrations of anisomycin.

Fig. S1.

A

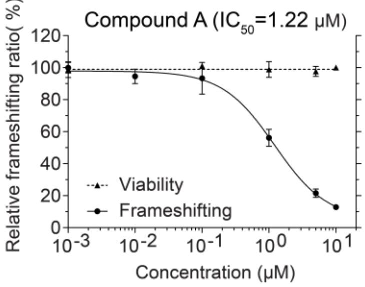

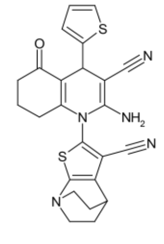

$C_{24}H_{21}N_5OS_2$

B

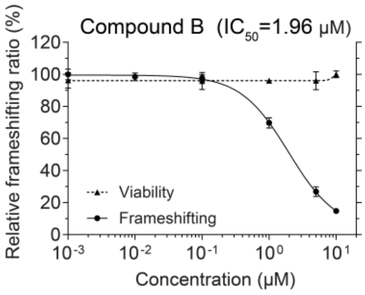

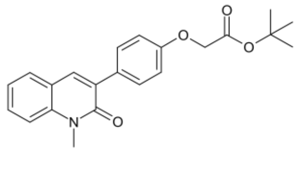

$C_{22}H_{23}NO_4$

C

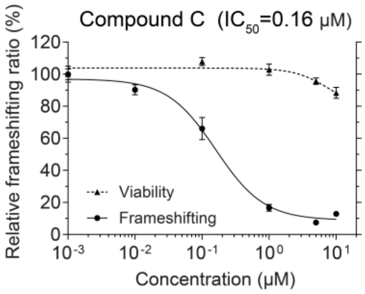

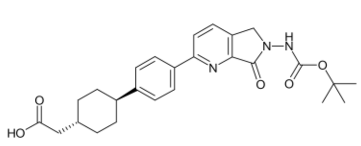

$C_{27}H_{33}N_3O_4$

D

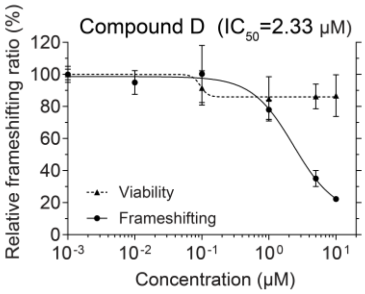

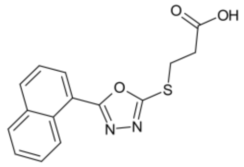

$C_{15}H_{12}N_2O_3S$

E

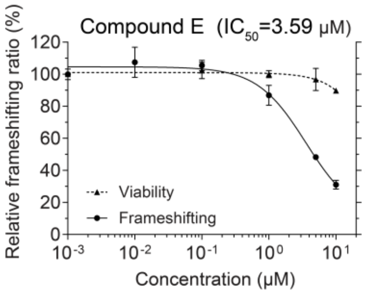

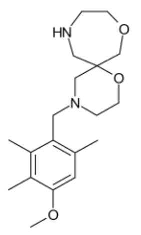

$C_{19}H_{30}N_2O_3$

Fig. S2.

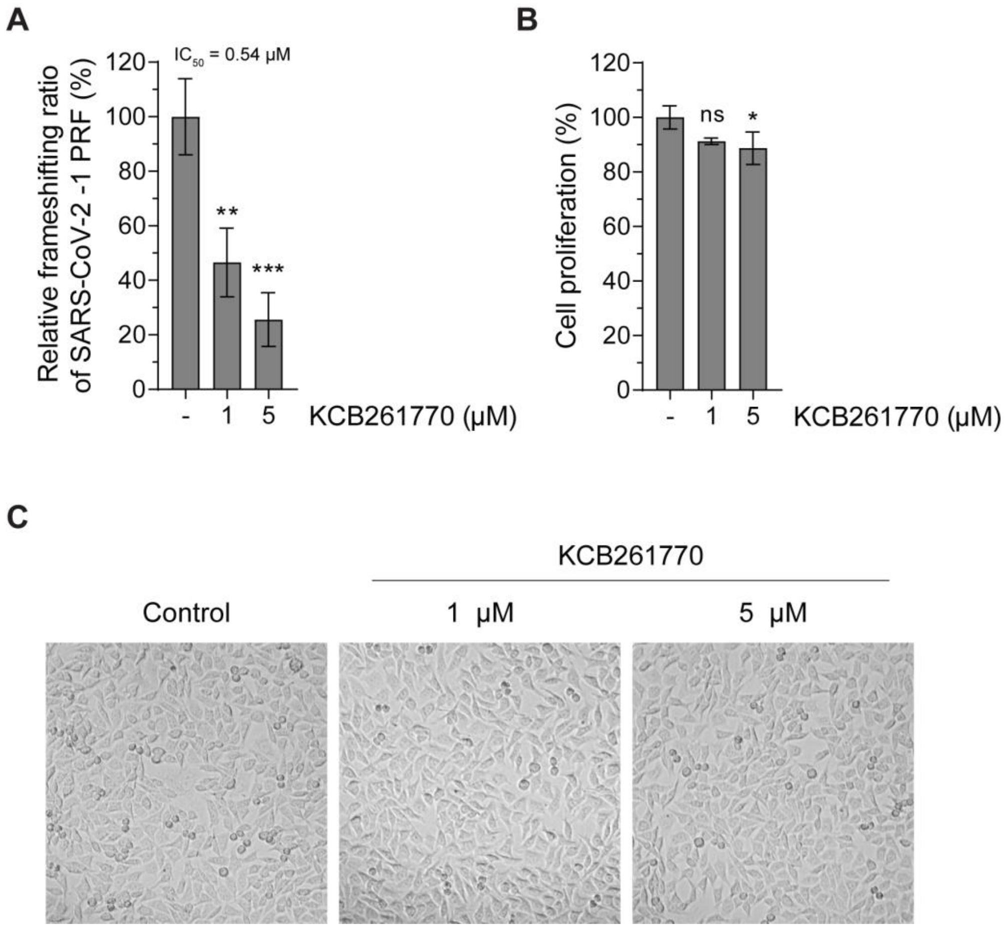
A
IC50 = 0.54 μM
Relative frameshifting ratio of SARS-CoV-2 -1 PRF (%)
120
100
80
60
40
20
0
**
***
-
1
5
KCB261770 (μM)
B
Cell proliferation (%)
ns
*
C
KCB261770
Control
1 μM
5 μM

Fig. S3.

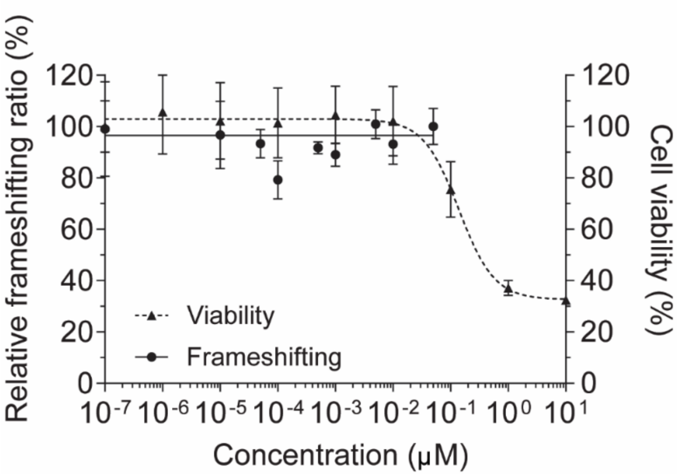

Relative frameshifting ratio (%)
120
100
80
60
40
20
0
Cell viability (%)
Viability
Frameshifting
10-7 10-6 10-5 10-4 10-3 10-2 10-1 10 0 10 1
Concentration (μM)

Table S1. Small molecules that have been reported previously.

Target	Chemical structure	Reference
HIV frameshift	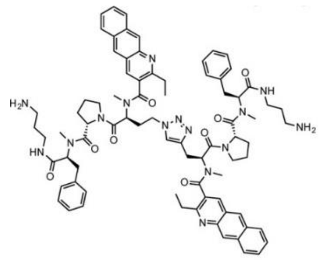<br>Triazole	Hilimire et al. (2017)
M_1 dsRNA virus and HIV frameshift	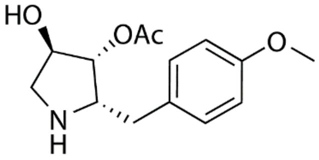<br>Anisomycin	Dinman et al. (1997)<br>Rakauskaite et al. (2011)
SARS-CoV-2 frameshift	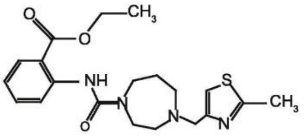<br>MTDB	Neupane et al. (2020)
SARS-CoV frameshift	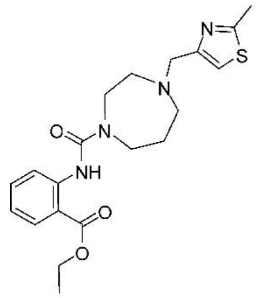<br>a novel compound, named 43	Park et al. (2016)
MERS-CoV frameshift	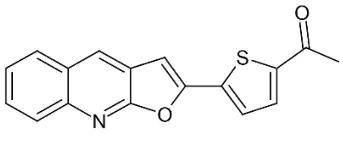<br>KCB261770	This study
